# Acute disseminated encephalomyelitis: a call to the clinicians for keeping this rare condition on clinical radar

**DOI:** 10.11604/pamj.2018.29.138.13942

**Published:** 2018-03-02

**Authors:** Liaqat Ali Chaudhry, Waseem Babur, Ghazala Aslam Chaudhry, Feddah Eid Al-Atawi, Asirvatham Alwin Robert

**Affiliations:** 1 Department of Internal Medicine, King Salman Military Hospital (NWAFH), Tabuk, Saudi Arabia; 2 Department of Family Medicine, King Salman Armed Forces Hospital (NWAFH), Tabuk, Saudi Arabia; 3 Department of Endocrinology and Diabetes, Diabetes Treatment Center, Prince Sultan Military Medical City, Riyadh, Saudi Arabia

**Keywords:** Encephalomyelitis, multiple sclerosis, acute onset encephalopathy

## Abstract

Acute disseminated encephalomyelitis is a rare disease of central nervous system, which can present with a variety of clinical manifestations. That is why first attack of ADEM, in particular remains a diagnostic puzzle. Early anticipation and diagnosis is important for better outcomes. We present a case of acute disseminated encephalomyelitis which initially had atypical clinical features with cough, expectoration, fever and later manifested strange neurological features, diagnosed to be a case of acute disseminated encephalomyelitis based on radio-imaging.

## Introduction

Acute disseminated encephalomyelitis (ADEM), an immune-mediated inflammatory demyelinating disorder, primarily affects the white matter of the brain and spinal cord [1, 2]. Acute onset encephalopathy expresses itself as distinctively self-limiting multifocal neurological deficits. This disorder is very similar to other acute demyelinating syndromes, like multiple sclerosis (MS), from the clinical and pathophysiological symptoms presented. However, ADEM is clearly detectable from other demyelinating disorders from its radio imaging MRI characteristics and laboratory reports. However, in the absence of any distinctive biomarker, definite diagnosis becomes challenging. Therefore, it is essential that such patients are followed up long term, as a few patients initially diagnosed with ADEM finally had MS [3, 4].

Most frequently, ADEM is heralded clearly by discernible febrile illness or immunizations. While ADEM is a monophasic disease, more often affecting pre-pubertal children (10-18yrs), MS is a chronic relapsing and remitting disease that attacks young adults. The demarcating lines between these different acute demyelinating diseases are blurred, except that the CSF results are less pronounced in ADEM. A clinical continuum is the more typical characteristic in the closely connected acute demyelinating conditions, including optic neuritis, hemiparesis with facial nerve palsy, transverse myelitis, meningoencephalitis, Guillain-Barré syndrome and vasculitis implicating the central nervous system. However, the primary pathophysiological processes involved continue to remain ambiguous [5]. In this study, the report of a 46-year-old male Saudi is included, who initially presented with a prodromal respiratory condition and only later on exhibited neurological signs and symptoms.

## Patient and observation

**Figure 1:**
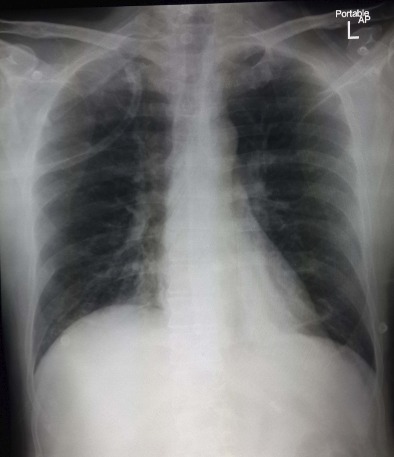
Chest X-ray revealed aberrations, showing bilateral discrete shadowing, similar to atypical pneumonia

A 46-year-old Saudi male smoker presented to the accident and emergency (A & E) room demonstrating symptoms of fever, intractable cough, mild expectoration and three days of feeling breathless. He came in after having spent time in Malaysia with his family 10 days prior. On examination, he appeared unwell, but was fully alert; his parameters included temperature 38°C, BP = 120/70, HR = 84, RR = 20 and O_2_ SAT = 87% at room air, correctable with 3 liters of oxygen. Chest auscultation revealed bilateral diffusely scattered crackles and rare wheezes. His chest x-ray (Figure 1) revealed aberrations, showing bilateral discrete shadowing, similar to atypical pneumonia. Other systemic examinations were unremarkable. His past history showed nothing relevant. He was then admitted to respiratory isolation with the provisional diagnosis of bilateral atypical viral pneumonia (H1N1 vs Corona-Virus) and respiratory insufficiency. Once the nasopharyngeal swabs were taken for viral studies, he was started on intravenous antibiotics and the oral antiviral tablet, Tamiflu. Initially, he responded well to the treatment. On day 4 of the treatment he was ambulant, afebrile and had normal range O_2_ saturation at room air. When his H1N1 and Co-Virus studies returned negative, the Tamiflu was stopped.

**Figure 2:**
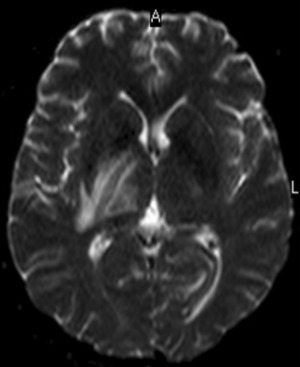
Magnetic resonance image of brain paraventricular area

**Figure 3:**
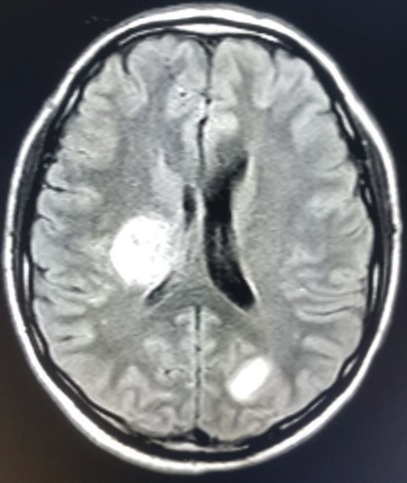
Magnetic resonance image of dorsolumber spine

However, the very next day the patient was reported to have altered sensorium, flaccid weakness on the left side of his body and facial palsy drooling of saliva; he also exhibited neck pain, preceded by urine retention and loss of sensation just below the level of the umbilicus a few hours prior. A Foley catheter was passed to relieve the urine retention. An urgent CT-Scan brain was arranged which highlighted a suspected edema/mass abutting the right ventricular wall, most probably ischemic in character; no mass effect was observed and a repeat CT-Scan or MRI of the brain and thoraco-lumbar spine was scheduled for 48 hours later. In light of these symptoms the treatment was revised; as he could not retain anything given orally, NPO (nil per os) a nasogastric tube (NGT) was passed for feeding, along with intravenous fluids and analgesics added, as well as to prevent aspiration. His carotid Doppler and echocardiography were normal, as were the coagulation profile and connective tissue panel. He was then transferred to the Intensive Care Unit (ICU) with desaturation, most likely arising from the aspiration of oropharyngeal secretions and necessitating high oxygen. His Glasgow Coma Scale (GCS) remained 15/15 and he continued to complain of neck and left shoulder pain. His power in the left upper limb registered 1/5 and left lower limb 0/5. Two days later he had right lower limb weakness as well, with 2/5 in power; his right upper limb was normal and the plantar reflexes were equivocal bilaterally. A revision was made of his treatment management under the impression of new stroke, associated with possible meningoencephalitis. He was then started on Aciclovir 200 mg IV q8h, besides inj. Meropenem 2 g IV q8h, vancomycin 1 g IV q12h, and analgesics. A lumbar puncture was done and the cerebrospinal fluid (CSF) drawn was sent for analysis. Next, an MRI of the brain and thoraco-lumbar spine was performed (Figure 2, Figure 3) on day 7 of admission.

### Laboratory investigations

The laboratory data were as given:

White cell count = 13.15, N = 92%, L = 6%, B = 0%, M = 1%, E = 1%, RBC = 4.04, Hb = 13, Hct = 35.9, mcv = 88.9, mch = 32.4, and plt = 314. The coagulation profile was normal; sodium = 137 meq, k = 4.1 meq, cl = 98 meq, BUN = 5.14 mmol/L, creatinine = 88 mmol/L, RBS = 5.6.1 mmol/L, HbA1C = 5.9%, C-reactive proteins = 3.66 mmol/L, Mag = 0.91 mmol/L, calcium = 2.09 mmol/L, T. protein = 71 g/L, S. albumin = 29 g/L, ALP = 79, AST = 23, ALT = 64, T. bilirubin = 6, cerebrospinal fluid analysis showed chloride = 120 mmol/L, glucose = 2.2 mmol/L, protein = 107+, color = clear. The WBC = 862/ul, RBCs = 165/ul, polymorphs = 95%, monomorphs = 5%, CSF other cells = 0%. The results of the cerebrospinal fluid culture and sensitivity revealed no growth and the India ink test returned negative while the Gram stain showed the presence of no bacteria, although several WBCS were present.

**Figure 4:**
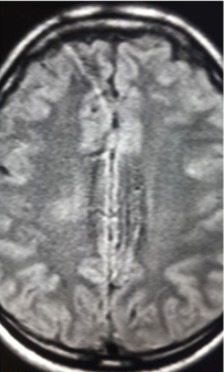
Computed tomography image of the patient brain

From the MRI brain (Figure 4), multiple bright areas (T2 W1 and Flair images) were observed in the right hypothalamus, covering a wide area upwards towards the region of the right basal ganglia, to right side of the brain stem and into the left occipital lobe. As these lesions revealed diffusion restriction on the DWI and were hypo-intense on the ADC map, the ischemic character of multiple brain parenchymal infarcts was evident. Although no hydrocephalus was observed, mild mass effect was visible on the right lateral and the 3rd ventricle. No proof of intracerebral bleed or extra-axial fluid collection was seen; normal calvarial bone and skull base were obvious. From the report of the MRI dorso-lumbar spine, bright foci were evident in the T2W1 of the distal thoracic spine at the D12 level, with minimal swelling of the medulla oblongata. Vertebral alignment and bone marrow intensity were normal.

In light of the findings of the MRIs of the brain and thoraco-lumbar spine, the treating team discussed the case. They considered this to be a diagnosis of acute disseminated encephalomyelitis (ADEM) and accordingly modified the patient´s treatment. They added inj. methylprednisolone and intravenous immunoglobulin IgG on day 8 of the admission. As he remained stable, the family requested that he be transferred to another tertiary hospital, the next day, because no neurological consultant was available at our facility. The same diagnosis was considered at the tertiary hospital and the identical line of management was performed. The patient began to show improvement.

## Discussion

Acute disseminated encephalomyelitis (ADEM) has been found to more frequently affect prepubertal children, from 8 to 18 years in age, and young adults, with an annual incidence of 0.4-0.8/100,000 of reported cases, predominantly occurring in the winter and spring seasons. In most instances, patients with this condition have been reported immediately after a prodromal recognizable infection, as in the case of the subject in this study, or following a recent immunization in the case of children [6, 7].

Characteristically, the ADEM symptoms start to present within one to eight weeks, as a reaction to an antigenic challenge. Although the typical and more common hallmark of this disorder is monophasic illness, in a minority instances a multiphasic course is manifested as well. This is dependent on the site and scope of the neuronal tissue affected. ADEM onset can occur suddenly, out of the blue as it were, or develop slowly over the duration of a few days or weeks, as was true for the patient in this study. The usual signs include the sudden and unexplained onset of multifocal localizing neurological symptoms like pain in the limbs and neck, focal or generalized seizures, psychosis, aphasia, dysphasia and visual field defects [8]. The patient in this study continuously complained of neck pain, preceded by urine retention, ataxia, facial palsy motor with drooling of saliva from the left side, sensory loss just below the level of the umbilicus and altered sensorium. Until other conditions like meningoencephalitis and demyelination disorders like multiple sclerosis (MS) are excluded, patients are most frequently treated empirically with a combination of antibiotics, antivirals and, at times, with corticosteroids for vasculitis.

Normally, cerebrospinal fluid (CSF) is unremarkable; however, sometimes it might reveal heightened protein and low glucose levels resembling bacterial meningitis but without any cellular constituents as supporting evidence for bacterial infection and negative cultures. This was the case for the patient in this study, who showed PCR (poly-chain reaction) on CSF as being negative for Mycobacterium tuberculosis. The IgG and IgM subtypes in the CSF at times are linked to ADEM and MS, although they are not diagnostic. Electroencephalograms (EEG) are non-specific, while the visual evoked potentials may show abnormality and delay. When specific biomarkers for ADEM diagnosis are completely absent, they are very often delayed and are based on the one hand on the exclusion of the likelihood of the other demyelinating and meningoencephalitis disorders and on the other hand on abnormal clinical reports and characteristics that are obvious on radio imaging [9].

As MRI facilities are now widely available, the frequency of diagnosing ADEM has increased. On the MRI T2 imaging, disseminated and multifocal lesions are clearly seen in the white matter implicating the basal ganglia, thalamus, brain stem and spinal cord. These lesions demonstrate the characteristics of edema, inflammation and demyelination [10, 11]. In the case of the patient in this study, the right paraventricular, brain stem, basal ganglia and thoracic (T6-8) regions were included. The CT-brain performed initially in this patient highlighted abnormal shadowing along the right paraventricular wall, completely covering the right ventricle, which the radiologist described as asymmetry of the ventricles resulting from edema or mass of ischemic nature; hemorrhage was absent and a repeat brain CT or MRI was recommended after 48 hours. In some instances, the initial radio imaging may be normal; therefore, a repeat radio imaging post 48 hours is essential, which may expose any abnormality more accurately and which is consistent with the diagnosis of ADEM, as in this patient.

With reference to the treatment for ADEM instances of patients improving without any treatment have been reported; however, the recovery may be partial in a few who did not receive any type of treatment, especially with corticosteroids or other immunomodulating drugs [12]. ADEM is usually treated with general supportive care, feeding via nasogastric tube (NGT) and implementation of aspiration precautions in patients experiencing swallowing difficulties, as in the case of the patient in this study. Preventive measures against deep vein thrombosis are taken, for patients with immobility and ataxia as well as preventive steps against falls and fractures. The first line treatment practiced, most commonly, until the present day, is the administration of high methylprednisolone doses (10-30 mg/kg/day x 3-5 days) given solely intravenously or together with either plasmapharesis or intravenous immunoglobulins in the more serious cases. The availability of intravenous immunoglobulins is the preferred treatment, which is equally effective when compared with plasmapheresis, which is not easily available and is technically more of a challenge. Tapering doses of oral corticosteroids are given for 4 to 6 weeks to avoid a relapse, after administering the initial intravenous high dose of methylprednisolone. While immunoglobulins (IgG) are given intravenously in doses of 0.4g/kg/day for 5 days, it has been found to be most effective when simultaneously administered with methylprednisolone or in cases where corticosteroids have failed.

When reliable and pathognomonic clinical proofs or biomarkers for patients diagnosed with ADEM are lacking, it becomes vital to exclude some disorders which are similar or have closely resembling characteristics. It is essential that primarily acute or chronic infective causes of meningoencephalitis must be excluded, as their treatment involves antimicrobial agents rather than immunomodulating agents. For the patient in this study, antiviral and antibiotics were used initially, following the impression of infective meningoencephalitis, particularly because of the persistent neck pain and CSF analysis reports. However, when the MRI and culture and poly chain reaction (PCR) reports were negative on the CSF for bacteria and mycobacterium respectively, the treatment was changed. One other significant differential diagnosis was MS, as it reveals a few specific CSF features like oligoclonal IgM bands apart from the absence of any prodromal febrile illness or immunization and presents neurological complaints which spread quickly, over time and space. Another condition, Guillain-Barré syndrome, exhibits predominantly ascending motor paraplegia with, at times, a Miller Fischer variant that follows in the wake of a febrile illness like gastroenteritis or infection of the respiratory tract.

Overall, the prognosis of ADEM is good in a substantial number of pre-puberty young patients, and ensures total recovery without recurrence [13]. This is the reason for ADEM, in such cases, to be referred to as a monophasic disorder. In the minority of cases, the patient may suffer a relapse, with a few symptoms, and is therefore termed multiphasic disseminated encephalomyelitis (MDEM) [14]. The recurrence of this disease in relatively young adults may represent a second attack of MS, after several months to years. It can be discerned by optic neuritis, oligoclonal bands in the CSF and the presence of periventricular lesions on the MRI. In such cases, early diagnosis of MS and prompt treatment is of immense prognostic value.

## Conclusion

Acute disseminated encephalomyelitis being a rare disease presenting with variable neurological manifestations and often preceded by a febrile illness must be kept in mind while looking for other important differential diagnosis. Early anticipation and use of radio-imaging like CT Scan or MRI of brain and spinal cord is of paramount importance for early diagnosis and prompt treatment of ADEM. Fortunately ADEM remains a monophasic condition in large majority of cases carrying good prognosis. Multiple sclerosis is a closely related other important diagnosis which needs to be confirmed and treated early. Especially, recurrence of neurological manifestations after months and years in such cases may represent a second attack of MS.
